# Machine Learning Insight: Unveiling Overlooked Risk Factors for Postoperative Complications in Gastric Cancer

**DOI:** 10.3390/cancers17071225

**Published:** 2025-04-04

**Authors:** Sejin Lee, Hyo-Jung Oh, Hosuon Yoo, Chan-Young Kim

**Affiliations:** 1 Department of Surgery, Jeonbuk National University Hospital, 20 Geonji-ro, Deokjin-gu, Jeonju 54907, Republic of Korea; mign5n@gmail.com; 2 Research Institute of Clinical Medicine of Jeonbuk National University-Biomedical Research Institute of Jeonbuk National University Hospital, Jeonju 54907, Republic of Korea; 3 Department of Library & Information Science, Jeonbuk National University, Jeonju 54896, Republic of Korea; ohj@jbnu.ac.kr; 4 Research Division for Data Analysis, Korea Institute of Science and Technology Information (KISTI), Daegu 41515, Republic of Korea; rhsblossom@naver.com

**Keywords:** gastrectomy, prediction of complication, machine learning algorithms

## Abstract

Background: Since postoperative complications after gastrectomy for gastric cancer are associated with poor clinical outcomes, it is important to predict and prepare for the occurrence of complications preoperatively. Conventional models for predicting complications have limitations, prompting interest in machine learning algorithms. Machine learning models have a superior ability to identify complex interactions among variables and nonlinear relationships, potentially revealing new risk factors. This study aimed to explore previously overlooked risk factors for postoperative complications and compare machine learning models with linear regression. Materials and Methods: We retrospectively reviewed data from 865 patients who underwent gastrectomy for gastric cancer from 2018 to 2022. A total of 85 variables, including demographics, clinical features, laboratory values, intraoperative parameters, and pathologic results, were used to conduct the machine learning model. The dataset was partitioned into 80% for training and 20% for validation. To identify the most accurate prediction model, missing data handling, variable selection, and hyperparameter tuning were performed. Results: Machine learning models performed notably well when using the backward elimination method and a moderate missing data strategy, achieving the highest area under the curve values (0.744). A total of 15 variables associated with postoperative complications were identified using a machine learning algorithm. Operation time was the most impactful variable, followed closely by pre-operative levels of albumin and mean corpuscular hemoglobin. Machine learning models, especially Random Forest and XGBoost, outperformed linear regression. Conclusions: Machine learning, coupled with advanced variable selection techniques, showed promise in enhancing risk prediction of postoperative complications for gastric cancer surgery.

## Simple Summary

This study aimed to improve the prediction of postoperative complications after gastrectomy for gastric cancer using machine learning (ML) models. Data from 865 patients who underwent surgery between 2018 and 2022 were analyzed, incorporating 85 variables, including clinical, laboratory, intraoperative, and pathological data. ML models were trained and validated (80/20 split) with steps for missing data handling, variable selection, and hyperparameter tuning. Using backward elimination and a moderate missing data strategy, the highest model performance was achieved (AUC 0.744). ML identified 15 key risk factors, with operation time, preoperative albumin, and mean corpuscular hemoglobin levels being the most predictive. Random Forest and XGBoost outperformed traditional linear regression. These findings suggest that ML, combined with refined feature selection, may enhance risk stratification for postoperative complications in gastric cancer surgery.

## 1. Introduction

Gastric cancer is a prevalent malignancy, significantly contributing to global cancer-related morbidity and mortality [1]. Surgical interventions are standard treatment options for this aggressive disease. However, postoperative morbidity rates have been reported to reach up to 46%, negatively impacting health outcomes and imposing a significant burden on healthcare resources [2]. The precise prediction of these postoperative complications is pivotal, leading to the development of many conventional models [3,4,5,6].

Recently, machine learning algorithms have been increasingly and widely applied across various domains of medicine, such as hybrid models for white blood cell classification and the prediction of maternal health risks [7,8]. In terms of predicting postoperative outcomes, machine learning has demonstrated superior performance, often outshining traditional statistical techniques [9,10,11,12]. Traditional statistical methods generally presuppose linear relationships and assign unchanging weights to risk factors. Such assumptions can inadvertently neglect complex interactions. Conversely, machine learning algorithms are adept at discerning, nonlinear associations and intricate dependencies, providing a more comprehensive and dynamic interpretation of data [13,14,15,16]. Despite the growing reliance on machine learning for predictions, many researchers still tend to focus on well-acknowledged variables, thereby possibly neglecting unexplored yet significant factors [16,17,18,19,20,21].

A truly comprehensive data-driven approach would ideally involve collecting large datasets from multiple medical centers [20,22]. However, given the constraints of time and cost, such an approach can be challenging. Recognizing these challenges, we initiated a preliminary study at one institution to uncover potential overlooked risk factors that could be revealed through machine learning models.

The primary goal of this study was to investigate the role of previously overlooked yet impactful variables in machine learning models. Furthermore, we aimed to determine if machine learning models, when trained in this context, could surpass predictions made through linear regression in varied situations. Our approach differs from prior research by employing a wide array of variables and applying systematic feature selection techniques to highlight clinically meaningful predictors. While most existing studies using machine learning have focused on well-established variables or imaging data [23,24], our study sheds light on routinely available preoperative parameters, such as CBC-related markers, that have rarely been emphasized. Insights from this study could serve as a foundation for future extensive multi-institutional data collection and refined research directions.

## 2. Material and Methods

### 2.1. Patient Selection

**Figure 1:**
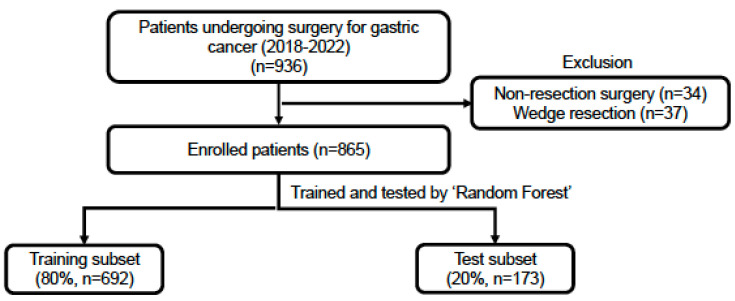
Study flowchart.

This study, approved by the local ethics committee (approval number: CUH 2023-07-010), retrospectively investigated the cohort of 936 patients who underwent gastric cancer surgery from January 2018 to December 2022 in single center. After the exclusion of 71 patients due to procedures like bypass surgery, wedge resection, and exploratory surgery, 865 patients remained for the machine learning analysis (Figure 1). All the surgical procedures were conducted according to the Korean gastric cancer treatment guidelines [25]. The Strengthening the Reporting of Cohort, Cross-sectional, and Case–Control Studies in Surgery (STROCSS) research guidelines were used to adhere to research standards [26]. This research also was retrospectively registered in ResearchRegistry.com.

### 2.2. Data Collection and Completeness

The dataset focused on data acquired pre-operation and intra-operation. However, postoperative pathological results that mirrored preoperative conditions were also incorporated. The collected 83 variables were categorized into preoperative, intraoperative, and postoperative pathologic variables. Preoperative variables comprised demographic and clinical data points, including blood and biochemical markers such as white blood cell count, hemoglobin, and carbohydrate antigen 19-9, among others. Intraoperative variables consisted of details about the surgery, such as the duration of operation, approach type, and tumor gross type. Postoperative pathologic variables included the pathologic TNM status in line with AJCC/UICC 8th edition [27] and tumor differentiation grade (Supplementary Table S1). Postoperative complications were defined as any adverse event that required additional pharmacologic, interventional, or surgical management within 30 days after surgery. All postoperative complications were graded according to the Clavien–Dindo classification at grade 1 or higher [28].

Variables are presented as numbers (percentages).

Of the 83 variables, 32 showcased missing values. Criteria for their inclusion or exclusion for analysis were based on the extent of absent data:

1. Over 30 missing values—Neutrophil percentage, Lymphocyte percentage, and 21 others, totaling 23 variables
2. Between 10 and 30 missing values—Total protein, Carcinoembryonic antigen, and Carbohydrate antigen 19-9, totaling 3 variables
3. Less than 10 missing values—Red blood cell distribution, Plateletcrit, Mean platelet volume (MPV), Platelet distribution width (PDW), Alkaline phosphatase, and Phosphorus, totaling 6 variables.

### 2.3. Machine Learning Methodology

To counteract data imbalance, we incorporated three sampling techniques: base, under-sampling, and over-sampling. Model efficacy was assessed using metrics such as accuracy, precision, recall, and area under the curve (AUC). The dataset was partitioned into 80% for training and 20% for validation. For enhanced evaluation precision, random resampling was performed 100 times.

We adopted three strategies to handle the 32 variables with missing data. Depending on the strategy, the resultant data varied in terms of the number of patients and variables retained.

1. Complete Case Strategy: All variables with any missing values were omitted, leaving us with data from all 865 patients but only 53 variables.
2. Moderate Missing Data Strategy: We removed variables with more than 10 missing values and then excluded patients with any missing data. This process left us with data from 847 patients and 59 variables.
3. Liberal Missing Data Strategy: We discarded variables with over 30 missing values and then excluded patients with any missing data. As a result, we retained the most variables, 62 in total, but were left with data from 799 patients.

Considering the risk of overfitting in machine learning, we implemented the backward elimination method for variable pruning. Techniques like Random Forest also assisted in gauging feature significance. Post variable selection, we fine-tuned our models using the Grid Search method for hyperparameter optimization. XGBoost and Random Forest were benchmarked against linear regression models. We performed 100 samplings for a thorough comparison.

### 2.4. Tools and Software

Comparative analyses concerning complications were executed using Pearson’s chi-square two-sided test and the t-test, facilitated by IBM SPSS 29. Statistical analyses were performed using Python (Version 3.8.10, Python Software Foundation), and the machine learning procedure was performed using the Python Sklearn package (Scikit-learn 1.2.0 for Random Forest, Multiple Linear Regression), XGBoost 1.7.5 for XGBoost).

## 3. Results

### 3.1. Postoperative Complications

A total of 222 patients had complications after gastric cancer surgery (25.7%). The rate of grade IIIa or higher complications was 8.9% (77 patients). Detailed incidence and type of postoperative complications are presented in Table 1.

### 3.2. Optimizing AUC in Model Development

In predictive modeling, the metric of AUC serves as an insightful measure of a model’s capability to distinguish between outcomes. Initially, the “Complete strategy” for missing data, when paired with “base” sampling, yields an AUC of 0.709. However, with “under-sampling” and “over-sampling”, the AUC fluctuates marginally to 0.707 and 0.712, respectively. The “Moderate strategy” exhibits a consistent AUC, ranging from 0.715 to 0.718 across different sampling techniques. The “Liberal strategy” shows a similar trend, with its AUC values hovering around 0.712.

During the variable selection process, the “Backward elimination” strategy, particularly when combined with the “Moderate strategy”, proved to be highly effective. This approach achieved AUC values exceeding 0.74, regardless of the sampling method used, indicating its consistent performance in various scenarios. The combination of “Hyperparameter tuning” with the “moderate strategy with backward elimination” also revealed impressive results. Using the “base” sampling yielded an AUC of 0.739, but with the “over-sampling” technique, this number increased slightly to 0.744, the highest recorded for this dataset (Table 2).

### 3.3. Interpretable Variables Selection

**Figure 2:**
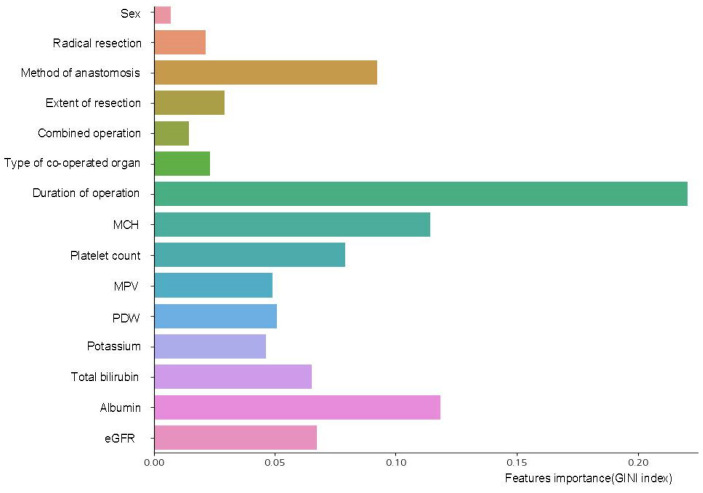
Identification of key variables via AUC optimization.

During AUC optimization in model development, variable selection emerged as a key factor in improving model performance. Through the backward elimination method, our study identified 15 pivotal variables: gender, radical resection, method of anastomosis, extent of resection, combined operation, type of co-operated organ, duration of operation, mean corpuscular hemoglobin (MCH), platelet count, MPV, PDW, potassium, total bilirubin, albumin, and estimated glomerular filtration rate (eGFR). These variables highlight their importance within the model, providing insights into their influence on outcomes (Figure 2). Notably, the duration of operation was the most impactful variable, followed closely by pre-operative levels of albumin and MCH. The method of anastomosis and platelet count were also influential. Among the selected variables, nine were pre-operative factors: gender, MCH, platelet count, MPV, PDW, potassium, total bilirubin, albumin, and eGFR. The remaining six variables, including radical resection, method of anastomosis, extent of resection, combined operation, type of co-operated organ, and duration of operation, were identified during surgery.

This graph illustrates the 15 critical variables pinpointed through the backward elimination method during AUC optimization. Each variable emphasizes its significance and impact on the model’s performance. Notably, the ‘Duration of operation’ emerged as the most influential, followed by pre-operative measurements of ‘albumin’ and ‘MCH’. Of these variables, nine were ascertained pre-operatively, and the remaining six were recognized during surgery.

The Gini Index is a measure of impurity. The probability of a randomly selected object from the target variable’s *i*th category being misclassified into the target variable’s *j*th category is P(*i*)P(*j*). Here, P(*i*) represents the probability that an object in each node belongs to the target variable’s ith category. Summing up these misclassification probabilities, we obtain

$$G=\sum_{i-1}^{c}\times \sum_{i+j}P\left(i\right)P(j)$$

which serves as an estimate of the misclassification probability under the given classification rule. In this context, c refers to the number of categories in the target variable.

Abbreviations: AUC; area under the curve, MCH; mean corpuscular hemoglobin, MPV; mean platelet volume, PDW; platelet distribution width, eGFR; estimated glomerular filtration rate.

### 3.4. Performance Comparison of Machine Learning Models with Conventional Multiple Linear Regression

**Figure 3:**
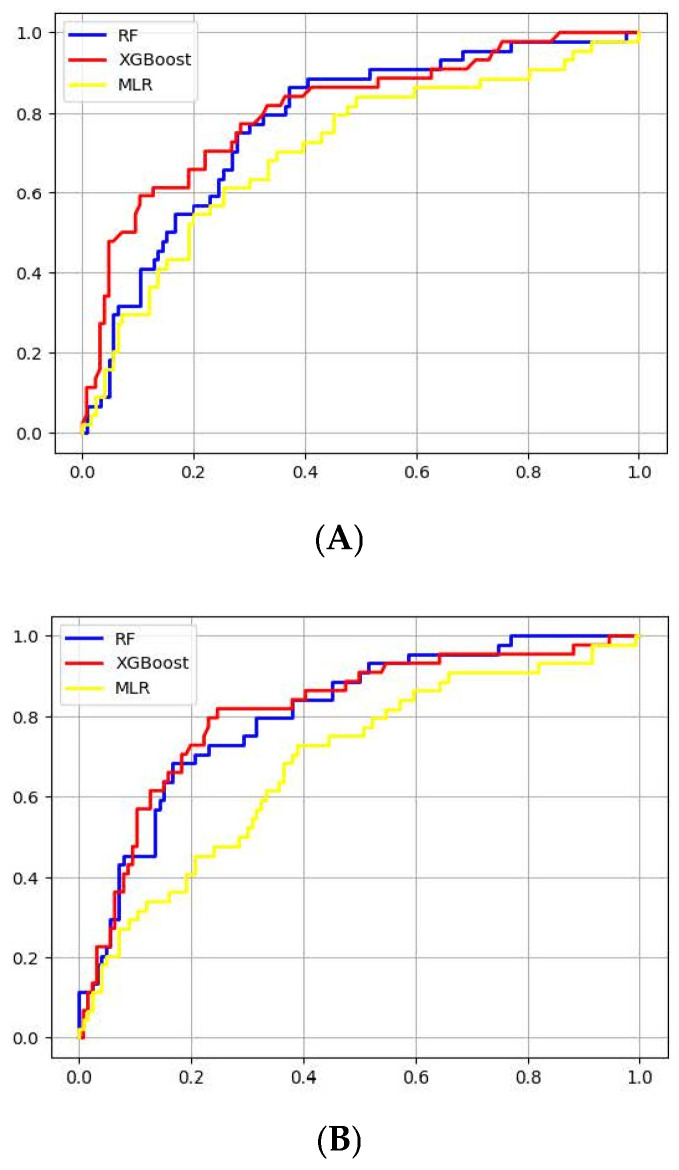
RF and XGBoost Models vs. MLR Performance Analysis: (**A**). Initial model comparison post-missing value treatment. (**B**). Performance post-variable selection using backward elimination. RF—Random Forest, MLR—Multiple Linear Regression.

The analysis compares the performance of three models, namely Random Forest (RF), XGBoost, and Multiple Linear Regression (MLR), using different variable strategies. Specifically, all variables under the ‘moderate strategy’ were considered, followed by backward elimination (Supplementary Tables S2 and S3). Using all variables post-cleanup, XGBoost outperformed the others in the test data with an AUC of 0.723 during over-sampling. RF and MLR followed closely, with their peak AUCs reaching 0.699 (Figure 3A). Upon variable selection, the test data revealed RF achieving the highest AUC at 0.744. XGBoost and MLR trailed with AUCs of 0.734 and 0.730, respectively (Figure 3B). While MLR serves as a foundational modeling approach, the ensemble methods, namely RF and XGBoost, exhibited enhanced performance in this study. Their enhanced performance can be credited to their ability to detect complex nonlinear relationships and combat overfitting through multiple decision trees or gradient-boosted stages. This performance advantage became even more evident when integrating efficient variable selection techniques, such as backward elimination.

## 4. Discussion

The evolution of risk prediction tools in surgery, from the American Society of Anesthesiologists classification in the 1940s to more contemporary tools, underscores the quest for better pre-operative patient assessment [4,5,6,29,30]. Despite the depth and breadth of tools available, the reliability of many remains questionable due to their inherent subjectivity and moderate predictive capacity [30].

This study aimed to utilize the capabilities of machine learning, taking advantage of its ability to identify complex data patterns that might be missed by traditional regression analyses. Where conventional statistical methods are bound by linear and additive assumptions and can potentially neglect key predictors, machine learning excels in detecting nonlinear relationships, providing crucial insights to improve data analysis [21,31].

A noteworthy discovery in our study was the significance of certain parameters in the Complete Blood Count (CBC) in predicting postoperative complications. In the field of gastric cancer, it may come as a surprise to many clinicians that detailed components of CBC can be so influential. Despite often being sidelined in routine assessments, parameters like MCH, MPV, and RDW were identified as key features in our machine learning models. When viewed through the lens of the literature, platelet count, MPV, RDW, and lymphocyte are inflammatory markers [32,33,34]. MPV, often linked to inflammatory processes across cardiovascular, respiratory, and neoplastic diseases, fluctuates based on the nature of the inflammation or disease [35,36,37]. The elevated MPV, often seen in cases of nutritional deficiencies or drug and alcohol usage, is strongly associated with increased mortality [38]. Studies have highlighted the significance of RDW and mean corpuscular volume (MCV) in patient outcomes, especially during surgical procedures. Abnormal RDW and MCV values can predict transfusion risks and postoperative outcomes in cardiac surgeries [39]. The relationship between RDW and mortality extends to older adults, regardless of their health conditions [40].

One intriguing observation for clinicians is that out of the 15 prognostic indicators identified, 9 can be readily ascertained preoperatively using basic demographic information and simple blood tests. Only six variables are derived intraoperatively. Surprisingly, the pathological results did not hold significant weight in the machine learning model. This suggests that if at-risk patient groups can be primarily identified through preoperative data, adjustments during surgery based on intraoperative variables might offer a strategy to mitigate postoperative complications.

From a technical standpoint in machine learning modeling, the technique lies not only in prediction accuracy but equally in adept feature selection. Striking a balance between a model laden with too many variables (which might cause overfitting) and one with too few (risking omission of crucial predictors) becomes critical. This delicate equilibrium, evident in our exploration of CBC parameters, highlights the nuanced finesse required in model creation. Incorporating tools like the Random Forest algorithm, known for its prowess in identifying patient characteristics, further bolstered our research. This algorithm, with its growing prominence in medical research, allows for a detailed dissection of risk factors, providing clearer insight into postoperative outcomes.

There are several limitations to this study, as outlined below:

1. Given that our research is preliminary, the significance of variables derived from our modeling cannot be fully ascertained with confidence.
2. As a result, validation through multi-institutional big data, particularly from high-volume tertiary centers [41], is imperative to reaffirm our findings.
3. The data are sourced from a single institution, and although comprehensive, they might not be representative of a broader patient population.
4. While we employed three strategies to handle missing values in the quest for an optimal predictive model, this approach may have inadvertently excluded variables that could be deemed significant based on clinical experience.
5. While we included essential model comparisons and variable selection strategies, more comprehensive ablation analyses—such as comparing the impact of different feature subsets or evaluating confusion matrices—were not fully explored. These analyses are crucial for evaluating model robustness and methodological innovation. To address this, a follow-up study is currently underway. This upcoming research will compare feature sets selected by AI-based backward elimination with those chosen by experienced surgeons using clinical expertise. We expect this future work to provide deeper insight into the clinical applicability and interpretability of machine learning models in surgical outcomes.

## 5. Conclusions

Our study underscores the transformative potential of machine learning in the realm of gastric surgery risk prediction. By unveiling previously underemphasized risk factors and emphasizing the nuanced value of CBC parameters, we pave the way for a new frontier in preoperative assessment. As the digital age progresses, so does the promise of leveraging advanced technology in refining our medical approaches. We believe our findings set the stage for expansive research, further harnessing the power of machine learning to enhance patient care in surgical settings.

## Figures and Tables

**Table 1:** Postoperative complications.

Variables	Value (N = 865)
Overall complication	222 (25.7)
Surgical complication	
Anastomotic leakage	30 (3.5)
Anastomotic stricture	3 (0.3)
Intra-abdominal bleeding	4 (0.5)
Intra-luminal bleeding	4 (0.5)
Fluid collection	9 (1.0)
Intra-abdominal abscess	2 (0.2)
Pancreatitis	6 (0.7)
Gastric stasis	19 (2.2)
Ileus	14 (1.6)
Intestinal obstruction	6 (0.7)
Fever	19 (2.2)
Wound	19 (2.2)
Other surgical complication	7 (0.8)
Medical complication	
Pulmonary	45 (5.2)
Renal	5 (0.6)
Urinary	8 (0.9)
Gastrointestinal	9 (1.0)
Hepatic	4 (0.5)
Other medical complication	9 (1.0)
Clavien–Dindo grade	
I	23 (2.7)
II	122 (14.1)
IIIa	42 (4.9)
IIIb	19 (2.2)
IV	4 (0.5)
V	12 (1.4)

Values in parentheses are percentages.

**Table 2:** Comparative analysis of missing data treatment and sampling on model performance metrics.

Step of Analysis	Missing Data Tx	Sampling	Train Accuracy	Test Accuracy	Test AUC	Test Precision	Test Recall
Missing data strategy	Complete strategy	Base	0.808 (0.783–0.824)	0.752 (0.717–0.780)	0.709 (0.593–0.838)	0.586 (0.000–1.000)	0.098 (0.000–0.182)
		Under	0.830 (0.792–0.871)	0.668 (0.572–0.751)	0.707 (0.597–0.803)	0.400 (0.313–0.508)	0.609 (0.364–0.864)
		Over	0.815 (0.775–0.841)	0.712 (0.642–0.786)	0.712 (0.558–0.797)	0.447 (0.318–0.590)	0.530 (0.318–0.773)
	Moderate strategy	Base	0.808 (0.793–0.829)	0.751 (0.712–0.788)	0.718 (0.601–0.789)	0.620 (0.000–1.000)	0.100 (0.000–0.205)
		Under	0.841 (0.793–0.891)	0.671 (0.582–0.735)	0.715 (0.604–0.796)	0.411 (0.319–0.493)	0.618 (0.455–0.818)
		Over	0.827 (0.792–0.853)	0.711 (0.629–0.788)	0.718 (0.589–0.797)	0.452 (0.327–0.611)	0.536 (0.364–0.750)
	Liberal strategy	Base	0.810 (0.789–0.826)	0.758 (0.725–0.800)	0.712 (0.630–0.825)	0.687 (0.000–1.000)	0.110 (0.000–0.244)
		Under	0.853 (0.807–0.883)	0.679 (0.606–0.769)	0.709 (0.611–0.824)	0.415 (0.333–0.550)	0.605 (0.415–0.805)
		Over	0.840 (0.792–0.870)	0.727 (0.650–0.850)	0.712 (0.623–0.852)	0.472 (0.340–0.707)	0.509 (0.293–0.756)
Backward elimination	Only moderate strategy	Base	0.799 (0.780–0.799)	0.747 (0.706–0.782)	0.741 (0.665–0.820)	0.562 (0.000–1.000)	0.099 (0.000–0.227)
		Under	0.807 (0.767–0.848)	0.675 (0.600–0.735)	0.738 (0.666–0.822)	0.424 (0.346–0.493)	0.694 (0.523–0.864)
		Over	0.799 (0.759–0.828)	0.710 (0.629–0.800)	0.741 (0.645–0.815)	0.459 (0.344–0.600)	0.649 (0.477–0.818)
Hyper parameter tuning	Only moderate strategy with backward elimination	Base	0.761 (0.747–0.783)	0.740 (0.718–0.759)	0.739 (0.663–0.814)	0.377 (0.000–1.000)	0.017 (0.000–0.091)
		Under	0.773 (0.741–0.813)	0.687 (0.629–0.759)	0.741 (0.659–0.841)	0.435 (0.368–0.526)	0.683(0.500–0.864)
		Over	0.767 (0.721–0.801)	0.711 (0.624–0.776)	0.744 (0.646–0.808)	0.461 (0.353–0.552)	0.657 (0.500–0.818)

The above result table summarizes the averages, maximums, and minimums of each metric after conducting 100 rounds of sampling. Abbreviations: AUC—area under the curve.

## Data Availability

The data presented in this study are available upon reasonable request from the corresponding author.

## Supplementary Materials

The following supporting information can be downloaded at: https://www.mdpi.com/article/10.3390/cancers17071225/s1, Table S1: List of Included Variables: Categorical and Numerical; Table S2: Performance Evaluation of Random Forest, XGBoost, and Linear Regression Models Across Different Sampling Techniques without variables selection; Table S3: Performance Evaluation of Random Forest, XGBoost, and Linear Regression Models Across Different Sampling Techniques with variables selection.
