# Supplementary material for: Machine Learning Insight: Unveiling Overlooked Risk Factors for Postoperative Complications in Gastric Cancer
Source: Cancers (Basel). 2025 Apr 4;17(7):1225. doi: 10.3390/cancers17071225 (PMC11987745; doi:10.3390/cancers17071225)
Supplement: Supplementary file 1 [file cancers-17-01225-s001.zip › cancers-3529691-supplementary.pdf]

Supplementary Table S1. List of Included Variables: Categorical and Numerical

**Preoperative variables**		Total (N=865)	Complication No (N=643)	Complication Yes (N=222)	P value
Sex	Male	610(70.5)	441(68.6)	169(76.1)	0.034
	Female	255(29.5)	202(31.4)	53(23.9)	
Age (years)*		65.0(11.4)	63.9(11.3)	70.0(11.0)	<0.001
Weight (kg)*		64.0(11.3)	64.3(10.9)	63.3(12.3)	0.253
Height (m)*		1.62(0.09)	1.63(0.09)	1.62(0.10)	0.359
BMI(kg/m2)*		24.2(3.4)	24.3(3.3)	24.1(3.7)	0.449
ASA score	1	83(9.6)	69(10.7)	14(6.3)	0.058
	2	604(69.8)	453(70.5)	151(68.0)	
	3	170(19.7)	115(17.9)	55(24.8)	
	4	8(0.9)	6(0.9)	2(0.9)	
Smoking	No	570(65.9)	430(66.9)	140(63.1)	0.446

	Current	138(16.0)	99(15.4)	39(17.6)	
	Quit<1yr	125(14.5)	88(13.7)	37(16.7)	
	Quit>1yr	32(3.7)	26(4.0)	6(2.7)	
Smoking(pack-years)*		8.6(16.1)	9.2(16.0)	10.9(16.3)	0.189
Alcohol drinking	No	540(62.4)	404(62.8)	136(61.3)	0.914
	Current	165(19.1)	121(18.8)	44(19.8)	
	Quit>1yr	160(18.5)	118(18.4)	42(18.9)	
Familial History of stomach cancer	No	732(84.6)	546(84.9)	186(83.8)	0.687
	Yes	133(15.4)	97(15.1)	36(16.2)	
Preoperative histology	High grade dysplasia	8(0.9)	7(1.1)	1(0.5)	0.156
	Papillary adenocarcinoma	2(0.2)	1(0.2)	1(0.5)	
	Well differentiated adenocarcinoma	166(19.2)	124(19.3)	42(18.9)	
	Moderate differentiated adenocarcinoma	288(34.5)	215(33.4)	83(37.4)	
	Poorly differentiated	224(25.9)	166(25.8)	58(26.1)	

	adenocarcinoma				
	Mucinous adenocarcinoma	4(0.5)	3(0.5)	1(0.5)	
	Signet ring cell	159(18.4)	126(19.6)	33(14.9)	
	Undifferentiated adenocarcinoma	1(0.1)	1(0.2)	0	
	Lymphoepithelioma-like gastric carcinoma	1(0.1)	0	1(0.5)	
	Neuroendocrine tumor	2(0.2)	0	2(0.9)	
Preoperative chemotherapy	No	853(98.6)	635(98.9)	218(98.2)	0.540
	Yes	12(1.4)	8(1.2)	4(1.8)	
White blood cell ($10^3/\mu L$)*		6.8(2.3)	6,8(2,2)	6,8(2,4)	0.896
Red blood cell($10^6/\mu L$)*		4.34(0.68)	4.40(0.62)	4.13(0.79)	<0.001
Hemoglobin(g/dL)*		13.2(2.3)	13.5(2.1)	12.4(2.7)	<0.001
Hematocrit(%)*		39.5(6.3)	40.2(5.6)	37.3(7.4)	<0.001
Mean corpuscular volume(fL) *		91.1(6.3)	91.3(5.9)	90.5(7.5)	0.101
Mean corpuscular hemoglobin(pg) *		30.4(2.8)	30.6(2.6)	30.1(3.3)	0.022

Mean corpuscular hemoglobin concentration(g/dL) *	33.4(1.5)	33.4(1.4)	33.1(1.6)	0.010
Red blood cell distribution width(%)*	13.4(2.1)	13.1(1.7)	14.1(3.0)	<0.001
Platelet(10^3/μL)*	251.0(86.8)	249.9(70.9)	254.3(121.9)	0.510
Plateletcrit(%)*	0.24(0.07)	0.24(0.06)	0.24(0.08)	0.821
Mean platelet volume(fL) *	9.73(0.85)	9.70(0.86)	9.81(0.82)	0.087
Platelet distribution width(fl)*	11.1(2.2)	11.2(2.3)	10.8(1.9)	0.031
Neutrophil percentage(%)*	58.8(11.1)	58.6(10.6)	59.2(12.1)	0.515
Lymphocyte percentage(%)*	30.6(9.9)	30.9(9.5)	29.9(10.9)	0.229
Monocyte percentage(%)*	7.7(2.5)	7.6(2.3)	7.9(3.1)	0.206
Eosinophil percentage(%)*	2.2(2.2)	2.2(2.2)	2.2(2.1)	0.959
Basophil percentage(%)*	0.6(0.3)	0.6(0.3)	0.6(0.3)	0.238
Lymphocyte count(10^3/μL)*	2.0(0.7)	2.0(0.7)	1.9(0.8)	0.218
Monocyte count(10^3/μL)*	0.5(0.2)	0.5(0.2)	0.5(0.2)	0.763
Neutrophil count(10^3/μL)*	4.1(2.0)	4.1(2.0)	4.1(2.2)	0.692
Eosinophil count(10^3/μL)*	0.1(0.2)	0.1(0.1)	0.1(0.2)	0.658

Basophil count($10^3/\mu L$)*	0.04(0.02)	0.04(0.02)	0.04(0.02)	0.505
Sodium(mmol/L)*	139.5(0.8)	139.7(2.7)	139.0(3.2)	0.003
Potassium(mmol/L)*	4.37(0.41)	4.36(0.40)	4.41(0.47)	0.136
Chloride(mmol/L)*	105.7(3.1)	105.7(3.0)	105.7(3.4)	0.760
Alkaline phosphatase(U/L)*	75.8(35.5)	76.5(38.5)	73.9(24.9)	0.348
Aspartate aminotransferase(U/L)*	28.2(24.9)	27.6(14.4)	29.9(41.9)	0.224
Alanine aminotransferase(U/L)*	26.6(21.5)	26.6(17.3)	26.4(30.6)	0.892
Total bilirubin(mg/dL)*	0.74(0.39)	0.75(0.36)	0.68(0.46)	0.014
Total Protein(g/dL)*	7.01(0.59)	7.07(0.54)	6.83(0.67)	<0.001
Albumin(g/dL)*	4.40(0.39)	4.45(0.36)	4.23(0.41)	<0.001
Blood urine nitrogen(mg/dL)*	16.5(7.9)	16.2(7.9)	17.2(8.0)	0.096
Creatinine(mg/dL)*	0.90(0.58)	0.88(0.58)	0.95(0.58)	0.121
estimated Glomerular filtration rate(mL/min/1.7)*	86.3(18.9)	87.7(17.8)	82.3(21.3)	<0.001
Uric acid(mg/dL)*	5.1(1.5)	5.1(1.4)	5.2(1.7)	0.293
Total calcium(mg/dL)*	9.5(0.5)	9.5(0.5)	9.3(0.6)	0.000

Phosphorus(mg/dL)*	3.4(0.5)	3.5(0.5)	3.4(0.5)	0.210
Cholesterol(mg/dL)*	175.6(41.9)	178.6(42.0)	166.3(40.3)	0.001
Triglyceride(mg/dL)*	134.0(84.5)	134.0(77.8)	133.9(99.7)	0.995
High density lipoprotein(mg/dL)*	48.3(13.1)	49.6(13.0)	44.6(12.7)	<0.001
Glucose(mg/dL)*	115.4(45.1)	112.8(38.4)	123.1(60.4)	0.007
Amylase(U/L)*	69.9(34.1)	69.3(33.9)	71.1(34.7)	0.668
Lipase(U/L)*	42.4(31.5)	40.7(22.8)	45.6(42.9)	0.209
Iron(µg/dL)*	71.1(50.9)	76.5(52.9)	62.8(47.1)	0.122
Unsaturated iron binding capacity(µg/dL)*	262.5(78.0)	264.7(75.5)	259.2(82.3)	0.688
Ferritin(ng/mL)*	104.7(128.6)	99.8(111.9)	120.5(171.5)	0.173
Total iron binding capacity(µg/dI)*	333.3(58.8)	341.2(54.4)	321.4(63.6)	0.053
C-reactive protein(mg/L)*	5.7(16.7)	5.5(16.0)	6.2(18.2)	0.725
Carcinoembryonic antigen(ng/mL)*	2.8(5.1)	2.3(3.3)	3.9(8.1)	<0.001
Carbohydrate antigen 19-9 (U/mL)*	34.6(231.9)	34.3(247.1)	35.3(180.3)	0.958

cT category	cT1	592(68.4)	464(72.2)	128(57.7)	<0.001
	cT2	78(9.0)	62(9.6)	16(7.2)	
	cT3	130(15.0)	83(12.9)	47(21.2)	
	cT4	65(7.5)	34(5.3)	31(14.0)	
cN category	cN0	688(79.5)	530(82.4)	158(71.2)	<0.001
	cN+	177(20.5)	113(17.6)	64(28.8)	
**Operative variables**					
Operator	1	79(9.1)	37(5.9)	42(18.9)	<0.001
	2	632(73.1)	509(79.2)	123(55.4)	
	3	78(9.0)	51(7.9)	27(12.2)	
	4	76(8.8)	46(7.2)	30(13.5)	
Duration of operation(min)*		137.2(63.1)	126.4(54.7)	168.7(74.5)	<0.001
Radical resection	Yes	820(94.8)	617(96.0)	203(91.4)	0.009
	No	45(5.2)	26(4.0)	19(8.6)	
Tumor gross type	EGC I	13(1.5)	10(1.6)	3(1.4)	<0.001

	EGC IIa	54(6.2)	47(7.3)	7(3.2)	
	EGC IIb	66(7.6)	53(8.2)	13(5.9)	
	EGC IIc	444(51.3)	350(54.4)	94(42.3)	
	EGC III	6(0.7)	3(0.5)	3(1.4)	
	Borrmann I	14(1.6)	10(1.6)	4(1.8)	
	Borrmann II	48(5.5)	29(4.5)	19(8.6)	
	Borrmann III	196(22.7)	127(19.8)	69(31.1)	
	Borrmann IV	24(2.8)	14(2.2)	10(4.5)	
Tubular location	Upper	101(11.7)	60(9.3)	41(18.5)	0.001
	Middle	311(36.0)	247(38.4)	64(28.8)	
	Lower	448(51.8)	332(51.6)	116(52.3)	
	Whole stomach	5(0.6)	4(0.6)	1(0.5)	
Circular location	Lesser curvature	392(45.3)	291(45.3)	101(45.5)	0.008
	Greater curvature	173(20.0)	140(21.8)	33(14.9)	
	Anterior wall	105(12.1)	78(12.1)	27(12.2)	

	Posterior wall	175(20.2)	125(19.4)	50(22.5)	
	Circumferential	20(2.3)	9(1.4)	11(5.0)	
Number of tumor	1	845(97.7)	627(97.5)	218(98.2)	0.557
	>1	20(2.5)	16(1.8)	4(2.3)	
Combined operation	No	667(77.1)	507(78.8)	160(72.1)	0.038
	Yes	198(22.9)	136(21.2)	62(27.9)	
Surgical approach	Open	110(12.7)	50(7.8)	60(27.0)	<0.001
	Laparoscopy	738(85.3)	579(90.0)	159(71.6)	
	Robot	17(2.0)	14(2.2)	3(1.4)	
Extent of resection	Distal	744(86.0)	573(89.1)	171(77.0)	<0.001
	Total	109(12.6)	65(10.1)	44(19.8)	
	Proximal	12(1.4)	5(0.8)	7(3.2)	
Type of reconstruction	Gastroduodenostomy	135(15.6)	92(14.3)	43(19.4)	<0.001
	Gastrojejunostomy	476(55.0)	378(58.8)	98(44.1)	
	Roux-en-Y anastomosis	242(28.0)	168(26.1)	84(33.3)	

	Esophagogastrostomy	6(0.7)	1(0.2)	5(2.3)	
	Double tract reconstruction	6(0.7)	4(0.6)	2(0.9)	
LN dissection	<D2	123(14.2)	88(13.7)	35(15.8)	0.444
	D2	742(85.8)	555(86.3)	187(84.2)	
Combined operation	No	785(90.8)	591(91.9)	194(87.4)	0.045
	Yes	80(9.2)	52(8.1)	28(12.6)	
Organ of combined operation	Gallbladder	56(6.5)	41(6.4)	15(6.8)	0.061
	Spleen	4(0.5)	2(0.3)	2(0.9)	
	Liver	3(0.3)	1(0.2)	2(0.9)	
	Colon	5(0.6)	3(0.5)	2(0.9)	
	Urologic	4(0.5)	1(0.2)	3(1.4)	
	Gynecologic	2(0.2)	1(0.2)	1(0.5)	
	Bile duct	1(0.1)	1(0.2)	0	
	Lung	2(0.2)	0	2(0.9)	
	Thyroid	1(0.1)	1(0.2)	0	

	Hernia	2(0.2)	1(0.2)	1(0.5)	
**Postoperative pathologic variables**					
Histology	Papillary adenocarcinoma	8(0.9)	5(0.8)	3(1.4)	0.215
	Well differentiated adenocarcinoma	113(13.1)	88(13.7)	25(11.3)	
	Moderate differentiated adenocarcinoma	301(34.8)	224(34.8)	77(34.7)	
	Poorly differentiated adenocarcinoma	256(29.6)	189(29.4)	67(30.2)	
	Mucinous adenocarcinoma	23(2.7)	12(1.9)	11(5.0)	
	Signet ring cell	147(17.0)	115(17.9)	32(14.4)	
	Undifferentiated adenocarcinoma	2(0.2)	1(0.2)	1(0.5)	
	Lymphoepithelioma-like gastric carcinoma	11(1.3)	7(1.1)	4(1.8)	
	Neuroendocrine tumor	4(0.5)	2(0.3)	2(0.9)	
pT category	T0	3(0.3)	3(0.5)	0	<0.001

	T1a	325(37.6)	261(40.6)	64(28.8)	
	T1b	255(29.5)	200(31.1)	55(24.8)	
	T2	69(8.0)	52(8.1)	17(7.7)	
	T3	141(16.3)	91(14.2)	50(22.5)	
	T4a	66(7.6)	35(5.4)	31(14.0)	
	T4b	6(0.7)	1(0.2)	5(2.3)	
pN category	N0	674(77.9)	525(81.6)	149(67.1)	<0.001
	N1	73(8.4)	48(7.5)	25(11.3)	
	N2	60(6.9)	37(5.8)	23(10.4)	
	N3a	45(5.2)	23(3.6)	22(9.9)	
	N3b	13(1.5)	10(1.6)	3(1.4)	
M category	M0	838(96.9)	627(97.5)	211(95.0)	0.068
	M1	27(3.1)	16(2.5)	11(5.0)	
TNM Stage	0	1(0.1)	1(0.2)	0	<0.001
	IA	547(63.2)	436(67.8)	111(50.0)	

	IB	75(8.7)	55(8.6)	20(9.0)
	IIA	74(8.6)	53(8.2)	21(9.5)
	IIB	48(5.5)	33(5.1)	15(6.8)
	IIIA	57(6.6)	31(4.8)	26(11.7)
	IIIB	27(3.1)	13(2.0)	14(6.3)
	IIIC	9(1.0)	5(0.8)	4(1.8)
	IV	27(3.1)	16(2.5)	11(5.0)

Variables are presented as number (percentage), *Numeric variables presents mean(SD)

Supplementary Table S2. Performance Evaluation of Random Forest, XGBoost, and Linear Regression Models Across Different Sampling Techniques without variables selection

	Model	Sampling	Accuracy	Precision	Recall	F1 score	AUC	AUC(95% CI)		MCC
Train	Random Forest	base	0.754	0.987	0.045	0.085	0.816	0.814	0.817	0.176
		under	0.757	0.812	0.669	0.733	0.861	0.859	0.864	0.523
		over	0.742	0.789	0.660	0.718	0.843	0.841	0.845	0.491
	XGBoost	base	0.774	0.695	0.172	0.271	0.787	0.780	0.795	0.271
		under	0.724	0.730	0.714	0.719	0.798	0.790	0.807	0.451
		over	0.733	0.722	0.757	0.737	0.809	0.802	0.816	0.468

	Linear regression	base	0.796	0.701	0.359	0.474	0.792	0.790	0.794	0.396
		under	0.744	0.767	0.701	0.732	0.821	0.818	0.824	0.490
		over	0.732	0.748	0.699	0.723	0.808	0.806	0.811	0.465
Test	Random Forest	base	0.742	0.285	0.010	0.019	0.699	0.691	0.707	0.033
		under	0.677	0.409	0.554	0.468	0.698	0.690	0.705	0.252
		over	0.702	0.436	0.493	0.461	0.699	0.691	0.707	0.259
	XGBoost	base	0.761	0.634	0.144	0.230	0.717	0.709	0.726	0.222
		under	0.681	0.432	0.685	0.526	0.719	0.711	0.726	0.328
		over	0.687	0.435	0.668	0.524	0.723	0.716	0.730	0.325
	Linear regression	base	0.754	0.553	0.279	0.367	0.697	0.689	0.705	0.258
		under	0.650	0.385	0.580	0.461	0.671	0.662	0.679	0.230
		over	0.678	0.411	0.555	0.471	0.683	0.675	0.690	0.254

To determine the confidence interval, the average value was calculated after 100 samplings.

Abbreviations: AUC, area under the curve; MCC, Mattews correlation coefficient

Supplementary Table S3. Performance Evaluation of Random Forest, XGBoost, and Linear Regression Models Across Different Sampling Techniques with variables selection

	Model	Sampling	Accuracy	Precision	Recall	F1 score	AUC	AUC(95% CI)		MCC
Train	Random Forest	base	0.761	0.979	0.070	0.129	0.820	0.819	0.822	0.221
		under	0.773	0.785	0.753	0.768	0.854	0.851	0.856	0.547

		over	0.767	0.770	0.763	0.766	0.843	0.841	0.845	0.535
	XGBoost	base	0.768	0.641	0.169	0.263	0.778	0.771	0.786	0.249
		under	0.721	0.725	0.715	0.717	0.788	0.780	0.797	0.446
		over	0.722	0.713	0.743	0.726	0.794	0.787	0.800	0.446
	Linear regression	base	0.771	0.630	0.265	0.372	0.605	0.603	0.608	0.297
		under	0.705	0.739	0.635	0.683	0.705	0.701	0.709	0.415
		over	0.704	0.735	0.636	0.682	0.762	0.759	0.765	0.411
Test	Random Forest	base	0.740	0.377	0.017	0.032	0.739	0.732	0.745	0.040
		under	0.687	0.435	0.683	0.529	0.741	0.734	0.748	0.332
		over	0.711	0.461	0.657	0.540	0.744	0.737	0.751	0.353
	XGBoost	base	0.763	0.642	0.158	0.246	0.727	0.717	0.736	0.233
		under	0.687	0.438	0.691	0.532	0.726	0.718	0.733	0.339
		over	0.696	0.447	0.694	0.541	0.734	0.727	0.741	0.351
	Linear regression	base	0.757	0.580	0.245	0.341	0.591	0.585	0.596	0.254
		under	0.708	0.452	0.607	0.517	0.675	0.668	0.681	0.322
		over	0.717	0.465	0.595	0.520	0.730	0.722	0.737	0.331

To determine the confidence interval, the average value was calculated after 100 samplings.

Abbreviations: AUC, area under the curve; MCC, Mattews correlation coefficient
